# Retraction Note: Fabrication of new composite NCuTiO_2_/CQD for photocatalytic degradation of ciprofloxacin and pharmaceutical wastewater treatment: degradation pathway, toxicity assessment

**DOI:** 10.1038/s41598-026-66316-4

**Published:** 2026-08-12

**Authors:** Roghayeh Noroozi, Mitra Gholami, Vahide Oskoei, Mohsen Hesami Arani, Seyedeh Azar Mousavifard, Binh Nguyen Le, Mehdi Fattahi

**Affiliations:** 1 Department of Environmental Health Engineering, Alborz University of Medical Sciences, Karaj, Iran; 2 Department of Environmental Health Engineering, School of Public Health, Iran University of Medical Sciences, Tehran, Iran; 3 School of Life and Environmental Science, Deakin University, Geelong, Australia; 4 Institute of Research and Development, Duy Tan University, Da Nang, Vietnam; 5 School of Engineering &Technology, Duy Tan University, Da Nang, Vietnam

Retraction of: *Scientific Reports* 10.1038/s41598-023-42922-4, published online 28 September 2023

The Editors have retracted this Article.

After publication, concerns were raised regarding the reliability of some of the reported data. Specifically, the scanning electron microscopy (SEM) image published in Fig 3b of this Article appears to closely resemble a SEM image in Fig 4b in a previously published paper by different authors^1^. In addition, concerns were raised with respect to the energy‑dispersive X‑ray spectroscopy (EDX) data. The Editors requested the underlying raw data, but the Authors were unable to provide it. The Editors therefore no longer have confidence in the findings reported in this Article.

Vahide Oskoei did not explicitly state if they agree or disagree with this retraction. Roghayeh Noroozi disagree with this retraction. The remaining Authors did not respond to correspondence from the Editors about this retraction.
